# Multicounty Outbreak of *Salmonella* Agbeni Linked to Ice in a Cooler at a County Fair — Illinois, August 2024

**DOI:** 10.15585/mmwr.mm7507a1

**Published:** 2026-02-26

**Authors:** Katherine E. Houser

**Affiliations:** 1 Brown County Health Department, Mount Sterling, Illinois.

**Summary**

**What is already known about this topic?**

*Salmonella* bacteria are typically spread through contaminated food or water or through contact with infected persons or animals.

**What is added by this report?**

In August 2024, at an Illinois county fair, 13 cases of *Salmonella enterica* serotype Agbeni infection (seven confirmed and six probable) were linked to ice that was contaminated during handling of beverage cans in a beer cooler; ice is an uncommon vehicle for *Salmonella* transmission at public events. Artificial intelligence–assisted tools helped synthesize relevant background information to support and contextualize the environmental health team’s assessment. All epidemiologic conclusions were based on traditional investigation methods, including case interviews, laboratory confirmation, and environmental findings.

**What are the implications for public health practice?**

Implementation of standardized, robust hygiene protocols for cooler sanitation and the handling of ice and beverages at events might prevent similar outbreaks.

## Abstract

On August 5, 2024, the Brown County (Illinois) Health Department (BCHD) was informed by the county sheriff that numerous potential jurors being screened for an upcoming trial had reported recently experiencing a gastrointestinal illness. One week later, on August 12, a laboratory-confirmed case of *Salmonella* *enterica* serotype Agbeni infection was reported to BCHD by the Illinois Department of Public Health. Investigation by BCHD identified seven laboratory-confirmed and six probable cases of *S.* *enterica* serotype Agbeni illness across five Illinois counties. All persons who became ill had attended the Brown County fair in rural Illinois during July 30–August 4 and reported drinking beer served from a cooler in the fair’s beer tent. No other common food or environmental exposures were identified. The cooler containing the beer was reused for multiple days and not cleaned. A generative artificial intelligence tool (ChatGPT 4.0, OpenAI; 2024) was used to assist with hypothesis generation during the investigation, supplementing traditional epidemiologic methods and contributing to identification of a shared, nonfood vehicle of transmission. This outbreak highlights the role of standardized hygiene protocols for cooler sanitation and beverage storage and handling at public events.

## Introduction

The Brown County Fair is held annually in Mount Sterling, Illinois, a rural, close-knit community of approximately 4,200 residents. The event attracts an estimated 36,000 attendees each year (approximately 5,000 per day). A majority of attendees view the evening events from a grandstand, which seats approximately 3,000 persons. The fair’s infield, a central gathering area reserved for premier ticket holders who may park flatbed trailers in the infield for tailgating and an optimal view of the main attractions, was situated in front of the main seating area in the grandstand. The infield also featured food vendors and a single beer tent; the beer tent was accessible only to attendees with wristbands documenting that they were of legal drinking age (21 years). The infield also included several portable restrooms and a limited number of portable handwashing stations. The 2024 fair was held July 30–August 4, with a beer tent staffed by a rotating committee of volunteers and fair board members, many of whom handled beverages and ice.

On August 5, the day after the fair ended, the county sheriff contacted the Brown County Health Department (BCHD) to report that during the screening of potential jurors for an upcoming trial, numerous candidates reported having recently experienced a gastrointestinal illness. Because of confidentiality restrictions, no names were provided, and at that time, no investigation was initiated. One week later, on August 12, the Illinois Department of Public Health reported to BCHD that a laboratory-confirmed case of *Salmonella* *enterica* serotype Agbeni, a nontyphoidal *Salmonella* species, had been identified. By the time the lab result was reported, the fair had concluded, and environmental sampling at the venue was not possible. This result, combined with the earlier reports of multiple cases of gastrointestinal illness among potential jurors, prompted an official outbreak investigation.

## Investigation and Results

### Case Definitions

CDC’s definition of a confirmed case of salmonellosis as a clinically compatible illness (e.g., diarrhea, abdominal pain, nausea, and sometimes vomiting) with laboratory isolation of a *Salmonella* species from a clinical specimen (*1*) was applied to all persons who became ill and had attended the fair. A probable case was defined as a clinically compatible illness epidemiologically linked to a confirmed case (e.g., shared exposure at the fair) but without culture confirmation, including a positive nonculture diagnostic method such as polymerase chain reaction testing without subsequent culture confirmation (*1*). This investigation was conducted as part of a public health response activity and was determined to be public health practice, not human subjects research; therefore, it was not subject to Institutional Review Board review.

### Patient Characteristics

Seven laboratory-confirmed cases of salmonellosis caused by *S.* Agbeni were identified through the National Electronic Disease Surveillance System among residents of five Illinois counties who had attended the fair (Table). In addition, six probable cases in close contacts of persons with laboratory-confirmed infection were also identified through patient interviews; these persons reported milder signs and symptoms (nausea and diarrhea) and did not seek testing or medical care. All 13 patients were aged 23–53 years. Ten cases occurred in men and three in women. Two of the six probable cases occurred in spouses of persons with confirmed cases; these persons reported similar, but milder, gastrointestinal symptoms.

**TABLE:** Characteristics of patients with confirmed or probable *Salmonella* Agbeni infection — Illinois, July–August 2024

Characteristic	No. (%)
**Total**	**13 (100)**
**Case status**	
Laboratory confirmed*	7 (54)
Probable^†^	6 (46)
**Sex**	
Female	3 (23)
Male	10 (77)
**Age group, yrs**	
<18	0 (—)
18–24	1 (7)
25–34	3 (23)
35–44	6 (46)
45–54	3 (23)
≥55	0 (—)
**Fair attendance date^§^**	
Jul 30	4 (31)
Jul 31	4 (31)
Aug 1	8 (62)
Aug 2	8 (62)
Aug 3	9 (69)
Aug 4	0 (—)
**Potential sources of exposure**	
Drank beer at the beer tent	13 (100)
Ate food from a specified vendor^¶^	9 (100)
Vendor A	2 (15)
Vendor B	3 (23)
Vendor C	2 (15)
Vendor D	1 (7)
Vendor E	5 (38)
Vendor I	1 (7)
Did not consume any vendor-purchased food	4 (31)
Used a portable toilet in the infield central gathering area, next to the beer tent	9 (69)
Used a permanent toilet inside a building	2 (15)
Attended livestock events	0 (—)
Sat in the grandstand seating area	0 (—)
Brought outside food or drink to the fair	0 (—)

* A clinically compatible illness with laboratory isolation of *Salmonella* species from a clinical specimen.

^†^ A clinically compatible illness epidemiologically linked to a confirmed case (e.g., shared exposure at the fair) but without culture confirmation, including a positive nonculture diagnostic method such as polymerase chain reaction testing, without subsequent culture confirmation.

^§^ Days are not mutually exclusive; percentages do not sum to 100 because some persons attended the fair on multiple days.

^¶^ Percentages do not sum to 100 because some persons purchased food from more than one vendor. Vendors F, G, H, J, K, and L are not listed because none of the persons with confirmed or probable cases ate food from these vendors.

### Potential Sources of Exposure

**Food, inadequate hand hygiene, and portable toilet use.** Initial hypotheses regarding infection source focused on foodborne illness linked to vendor-prepared meals or possible contamination from the communal portable toilets. All 13 persons who became ill were interviewed using a foodborne illness questionnaire provided by the Illinois Department of Public Health. Although nine persons who became ill reported that they had purchased and eaten food from one or more of six different vendors on different days, four patients had not eaten at the fair at all. Two patients reported that they cleaned their hands using their own personal hand sanitizer, and 10 patients reported that they did not wash their hands. Four patients reported never having used the portable toilets. None of the persons who became ill sat in the grandstand or worked in the livestock barns. However, all 13 persons who became ill reported 1) spending time in the infield area and 2) drinking canned beer from the beer tent. No illnesses were identified among persons who did not access the beer tent.

**Ice in the beer cooler.** BCHD staff members conducted interviews with persons who had worked at the beer tent during the fair to ascertain how the cooler and beer cans were handled. Fair organizers reported that the large improvised cooler in the beer tent consisted of a 10-ft length of non–food-grade corrugated black plastic farm drainage tile with four internal compartments. The cooler was only meant to contain ice and cans of beer. The ice for the fair was provided by a single local company and produced from a municipal water supply, and all fair vendors had access to the same ice for use in their vendor stalls. The cooler was rinsed out with a hose once at the beginning of the week. The cooler was not rinsed again and was never cleaned with soap, and no means for monitoring the cooler’s internal temperature was available. Staff members handled the ice and cans with their bare hands. Handwashing stations were not available inside the beer tent. Cans were kept submerged in ice, and the melted ice was replenished daily. The cooler was designed to drain through a single spigot; however, standing water was reported, suggesting incomplete drainage. Different staff members worked at the beer tent each night, including two persons who later received test results positive for salmonellosis; both reported drinking beer at the beer tent and were the only staff members who reported illness. The standing water in the cooler was not reported until after the fair had concluded and was not available for testing, nor was the cooler itself. No standardized cleaning or sanitization procedures for the cooler were reported.

The BCHD food inspector confirmed with BCHD communicable disease investigative staff members that ice can become contaminated if handled or stored improperly, consistent with the Illinois Food Code (77 Ill. Adm. Code 750.100: definition of contaminated food, and 750.330: protection from contamination after receiving), which recognizes ice as food and subject to contamination risks (*2*). A 2013–2023 systematic review documented contamination of food ice with enteric bacteria and viruses (e.g., coliforms and *Escherichia coli*, *Staphylococcus aureus*, *Vibrio* spp., and norovirus) and fungi, most often linked to inadequate ice-machine sanitation and handling; contamination was more common in ice produced on-site in food businesses than in industrially manufactured ice, supporting ice as a plausible contributor to cross-contamination and gastrointestinal outbreaks (*3*).

### Patient Interviews

Some interviewed attendees were hesitant to provide details because they did not want to implicate other members of their community. One patient with laboratory-confirmed salmonellosis reported observing leftover food stored overnight in the ice cooler on August 1. Fair board members stated that this practice was not permitted. Because the investigation was retrospective, the report could not be independently verified; however, unauthorized food storage in ice used for beverages represents a potential mechanism for cross-contamination.

### Measures to Identify Additional Cases

Because the community was small and close-knit, health department staff members attempted to identify additional cases by contacting personal acquaintances and monitoring social media posts and photos to identify persons who had attended the fair or visited potential areas of exposure. During August 12–21, health department staff members contacted fair board members and persons who they knew had attended the fair to ascertain whether these persons had visited the beer tent and whether they or persons they knew had become ill. No additional cases were identified.

### Artificial Intelligence Hypothesis Generation

Drinking beer from the fair’s beer tent was the only common exposure reported among all persons who became ill. Because the investigation began >1 week after the fair concluded, the ice and water were no longer available for testing, nor was the cooler. In the absence of other common food or environmental exposures, contamination of the ice used for beer storage was considered a plausible source for the outbreak. A generative artificial intelligence (AI) large language model (ChatGPT 4.0, OpenAI; 2024) was used to generate hypotheses of other possible sources of exposure by reviewing exposure patterns and identifying potential transmission pathways, supplementing the ongoing traditional epidemiologic investigation. No other common exposure sources were identified. On August 21, information regarding which days each fair attendee became ill, the vendors from whom they purchased food, what each person ate and drank, and whether the patients were in the infield or the grandstand was entered into the AI model, followed by a series of questions.

The first question entered into the AI model was, “Will *S.* Agbeni grow in an improperly drained cooler?” ChatGPT responded that *S*. Abegni could grow in a cooler under conditions that are favorable to *Salmonella* growth, including standing meltwater combined with hot summer days and lack of sanitation. The second question was, “Are any other sources, other than ice, likely if only canned beverages and no foods were available at this location?” The AI response indicated that *Salmonella*-contaminated meltwater contacting the exterior of beverage cans, followed by hand-to-mouth transfer of bacteria, was a plausible route of exposure. The third question was, “What is the likelihood that these infections occurred through contaminated ice rather than another exposure?” The AI response compared alternative hypotheses and indicated that, given the environmental conditions described and that no common food was identified, ice contamination was the most consistent explanation. The fourth question was, “Why would some persons become ill whereas others remained well?” The AI response suggested that variation in exposure dose, differences in how persons handled the cans, and fluctuations in the water temperature throughout the day might account for the distribution of illnesses. The fifth question was, “What examples of similar outbreaks have been documented in scientific literature?” The AI response identified studies in which contaminated ice or beverages stored in ice were linked to outbreaks of enteric illnesses (*3*–*5*). The final question entered into the AI model was, “What is the probability of a *Salmonella* outbreak linked to contaminated ice?” The AI response did not provide a definitive number but emphasized that such outbreaks are well-documented in the literature and represent a credible and likely source in this setting. The AI response further suggested that ice contamination is often an overlooked transmission vector, which might explain why precise probabilities were difficult to establish despite recurring evidence of its role in enteric outbreaks.

## Public Health Response

In response to the outbreak, BCHD developed specific guidance on cooler sanitation and safe beverage handling practices (BCHD, unpublished document, 2024). Educational materials were distributed to all participating vendors, event organizers, and community partners. In addition, BCHD contacted fair board members, food handlers, and volunteers, emphasizing proper hygiene practices and safe food storage methods. BCHD required routine sanitization of ice coolers using a bleach solution, and equipment used for beverage service remains subject to inspection and might be restricted from use if sanitation standards are not met. Interagency communication was strengthened through coordinated meetings and follow-up planning sessions to promote a unified prevention strategy for future events. BCHD provides ongoing surveillance during large public gatherings and incorporates food safety audits and random checks as part of routine event health inspections. Cooler sanitation protocols are now a required component of future vendor licensing agreements.

## Discussion

*S. enterica*, a leading cause of bacterial foodborne illness in the United States, is commonly associated with contaminated meat, produce, eggs, and other high-risk food items (*6*), including dairy products, precooked foods held warm, soups, stews, gravies, sauces, deli meats and sliced ready-to-eat meats, hot dogs and processed meats, seafood and shellfish, sushi and raw fish products, cut melons, cut tomatoes, cut leafy greens, raw sprouts, prepared salads, and garnishes. In this outbreak of *S. *Agbeni among attendees at a county fair, persons who became ill had purchased food from different vendors on different days, and four patients had not eaten at the fair at all, suggesting that an exposure other than food was the source of infection. The outbreak was linked to ice water used to store and chill beverages, which was likely contaminated. Transmission through improper ice- or beverage-handling practices is rarely reported (*4*). Because all the ice used at the fair was produced by a single local vendor, used a municipal water supply, and was distributed uniformly to all vendors, contamination at the source was considered unlikely. A more plausible explanation is that the ice became contaminated secondarily through improper handling practices, such as direct contact with contaminated food stored in coolers or via fecal-oral transmission from inadequate hand hygiene. Use of improvised beverage storage equipment that could not be adequately drained likely contributed to this outbreak; consequently, routine sanitization of ice coolers is now required. These pathways are consistent with known transmission routes for *Salmonella* spp. and highlight the importance of strict food safety and hygiene protocols during mass gatherings. Although consumption of contaminated food is the most common route for the spread of *Salmonella*, this investigation highlights the importance of considering uncommon exposures (*7*).

Hypothesis generation using AI helped identify contaminated ice as the most likely source. Although this technique did not follow a traditional surveillance protocol, AI was effective in this rural setting for rapid situational awareness and early case finding, especially because formal case reporting was delayed or limited. Clean ice that becomes contaminated has been implicated in outbreaks involving norovirus, *Cryptosporidium*, and *E. coli* (*4*), although *Salmonella* bacteria are rarely transmitted in this way (*5*).

### Limitations

The findings in this report are subject to at least three limitations. First, the delay between the end of the fair and the initial report of illness made identifying additional cases challenging. Second, testing of the cooler for contaminants was not possible, which precluded definitive identification of ice in the cooler as the source of the outbreak. Because environmental sampling was not possible, a generative AI tool (i.e., Chat GPT) was used only to summarize previously published outbreak investigations and environmental health literature to contextualize and support the environmental health team’s independently developed hypothesis. AI was not used for case finding, exposure assessment, or primary data analysis. Given the inherent limitations of generative AI tools, including potential inaccuracies and lack of source transparency, all AI-generated summaries were critically reviewed and validated against primary literature before incorporation. Finally, mild illness and limited awareness of reportability likely contributed to the underreporting of cases.

### Implications for Public Health Practice

This investigation underscores the importance of local adaptability and collaboration with event organizers, members of the community, and public health departments. In a small community, monitoring social media posts and photos, as well as personally contacting fair board members and persons who health department staff members had encountered at the fair, contributed to rapid situational awareness and early case finding but also contributed to reluctance to report, for fear of implicating a friend or neighbor as contributing to the outbreak. This outbreak highlights the role of implementing and enforcing food sanitation and hygiene practices, including proper handling and storage of ice, frequent cleaning of coolers, and prevention of cross-contamination to prevent similar outbreaks in comparable community event settings.

## References

[1] CDC. National Notifiable Diseases Surveillance System (NNDSS): Salmonellosis (*Salmonella* spp.) 2017 case definition. Atlanta, GA: US Department of Health and Human Services, CDC; 2017. https://ndc.services.cdc.gov/case-definitions/salmonellosis-2017/

[2] Illinois Register. Department of Public Health. Notice of adopted amendments: food service sanitation code (77 Ill. Adm. code 750). Springfield, IL: Illinois Department of Public Health; 2024. https://www.idph.state.il.us/rulesregs/2013_Rules/Adopted/77_IAC_750_12-6.pdf

[3] Triggiano F, Apollonio F, Diella G, Marcotrigiano V, Caggiano G. State of the art in hygienic quality of food ice worldwide: a ten-year review. Microorganisms 2024;12:690–709. 10.3390/microorganisms12040690 38674635 PMC11051916

[4] Jalava K, Kauppinen A, Al-Hello H, Räsänen S. An outbreak of norovirus infection caused by ice cubes and a leaking air ventilation valve. Epidemiol Infect 2018;147:e57. 10.1017/S095026881800314X 30501678 PMC6518581

[5] Scallan E, Hoekstra RM, Angulo FJ, Foodborne illness acquired in the United States—major pathogens. Emerg Infect Dis 2011;17:7–15. 10.3201/eid1701.P11101 21192848 PMC3375761

[6] CDC. *Salmonella* infection (salmonellosis). Atlanta, GA: US Department of Health and Human Services, CDC; 2023. https://www.cdc.gov/salmonella/

[7] World Health Organization. *Salmonella *(non-typhoidal). Geneva, Switzerland: World Health Organization; 2018. https://www.who.int/news-room/fact-sheets/detail/salmonella-(non-typhoidal)

